# Compliance with continuous positive airway pressure (CPAP) therapy for obstructive sleep apnea among privately paying patients- a cross sectional study

**DOI:** 10.1186/1471-2466-14-188

**Published:** 2014-11-29

**Authors:** Syed Fayyaz Hussain, Muhammad Irfan, Zeeshan Waheed, Naveen Alam, Saba Mansoor, Muhammad Islam

**Affiliations:** Department of Medicine, Kettering General Hospital, Kettering, UK; Department of Medicine, The Aga Khan University Hospital, PO Box 3500, Stadium Road, Karachi, 74800 Pakistan; Department of Medicine, Armed Forces Hospital Southern Region, Khamis Mushayt, Saudi Arabia; Department of Community Health Sciences, The Aga Khan University Hospital, Karachi, Pakistan

**Keywords:** Obstructive sleep apnea, Continuous positive airway pressure therapy, Compliance, Side effects, Pakistan

## Abstract

**Background:**

To evaluate the compliance, benefits and side effects associated with continuous positive airway pressure (CPAP) therapy among Pakistani patients treated for obstructive sleep apnea (OSA) in private sector.

**Methods:**

Patients diagnosed to have OSA based on overnight study who were recommended for CPAP therapy, between 1998 and 2003, were evaluated by telephonic survey and review of hospital notes. Compliance, benefits and side effects associated with CPAP therapy were assessed.

**Results:**

Out of 135 patients who were prescribed CPAP therapy, 75 could be contacted. Sixty (80%) started using CPAP within one month of diagnosis and 46 (61%) continued to use it long-term (beyond one year). Compliance with CPAP therapy was associated with higher body mass index, higher Epworth sleepiness scale score, history of witnessed apnea, and reduction in daytime sleepiness with CPAP therapy. OSA severity as assessed by apnea-hypopnea index did not affect compliance with CPAP therapy. Use of anti-depressants and CPAP induced sleep disturbances were associated with poor compliance with CPAP therapy.

**Conclusions:**

Obesity, excessive daytime sleepiness, witnessed apnea and improvement of daytime symptoms following use of CPAP were predictors of improved compliance. Use of antidepressants and CPAP induced sleep disturbances were predictors of poor compliance.

## Background

Obstructive sleep apnea (OSA) is relatively common disorder with estimated prevalence of 4% in men and 2% in women [1]. OSA is characterized by intermittent obstruction of the upper airway during sleep. Patients experience repetitive episodes of apnea (complete cessation of airflow), hypopnea (partial reduction in airflow) and oxygen desaturation, and sleep fragmentation. Common symptoms are of loud habitual snoring, frequent nocturnal awakening, unrefreshing sleep and excessive daytime sleepiness (EDS). Age, gender, obesity, and nasal blockage are some of the well-established risk factors for OSA [2]. OSA is associated with poor sleep quality, impaired cognition and increased risk of accidents [3]. Furthermore, there is increased risk of fatal and non-fatal cardiovascular events and the treatment of OSA favorably reduces the risks associated with OSA [4]. Similarly, OSA is associated with an increase in blood pressure that is improved with treating apnea but not by supplemental O2 therapy suggesting mechanisms other than hypoxemia are responsible for hypertension [5].

Continuous positive airway pressure (CPAP) therapy is the treatment of choice for OSA. CPAP therapy produces improvements in daytime sleepiness and cognitive function with improved quality of life compared with control [6]. Good compliance with CPAP therapy has been shown to achieve cardiovascular and metabolic benefits [7].

Despite the high efficacy of CPAP, treatment effectiveness is limited by variable adherence to prescribed therapy [8]. Failure to comply with CPAP therapy may occur in up to 25% to 50% patients, with patients typically abandoning therapy within the first 4 weeks of treatment [9]. In one review up to 91% of the patients discontinued the CPAP treatment in the first three years of therapy [10].

The aim of this study was to evaluate the compliance, benefits and side effects associated with CPAP therapy among Pakistani patients treated in private sector.

## Methods

The study was conducted at The Aga Khan University Hospital, a private teaching hospital in Karachi, Pakistan. Patients were diagnosed to have OSA based on suggestive clinical features (habitual snoring, witnessed apnea, unrefreshing sleep and excessive daytime sleepiness) and confirmed by an overnight-supervised oximetry or polysomnography. Two levels of sleep studies were performed; overnight full polysomnography in 55 (73.3%) and overnight pulse oximetry in 20 (26.7%) patients. The variables monitored in polysomnography were electroencephalogram (EEG), electrooculogram (EOG), chin and leg electromyogram (EMG), electrocardiogram (ECG), oronasal airflow (thermistor), oxygen saturation, and chest and abdominal wall movements (respiratory inductive plethysmograph). In overnight oximetry data was analysed for number of Oxygen (O_2_) desaturations, basal O_2_ saturation, minimum O_2_ saturation, cumulative time spent below 90% saturation and O_2_ desaturation index (total number of O_2_ desaturation divided by duration of recording in hrs). Waveform was recorded for the whole duration of recording as a compact one-page tracing and in an expanded hourly format for detailed review. Daytime sleepiness was assessed using Epworth sleepiness scale (ESS) score [11]. Threshold for treating OSA was apnea-hypopnea index (AHI) > 15 per hour or 3% oxygen desaturation index > 15 per hour accompanied with daytime symptoms. Nasal CPAP was initiated following a supervised titration study. Patients had a session on mask application and machine use. The patients had to buy their own machine.

The survey was done from January to March 2006. Patients diagnosed to have OSA between 1998 and 2003 were included in the study. A trained research officer reviewed their notes and contacted patients by telephone. As this was the telephonic survey, verbal consent was taken from all participants. Data was entered using a structured questionnaire that elicited information about symptoms, past medical history, drug history, family history, sleep history, examination findings and results of investigations. Compliance was assessed by asking multiple questions on daily day time and night time usage in hours and period of failure to use of CPAP machine. Benefits and side effects associated with CPAP therapy were also assessed.

Improvement in ESS score of two points or more was considered significant. Compliance was defined as continued (long-term) use of CPAP therapy beyond one year. Current smoker was defined as someone who continues to smoke any amount of tobacco either regularly or occasionally. A non-smoker was one who has never smoked and an ex-smoker was someone who had smoked in the past but had now stopped.

The study was approved by Ethics Review Committee (ERC) of Aga Khan University.

### Statistical analysis

The Statistical Package for social science SPSS version 16.0 was used for data analysis. Descriptive analysis was done for demographic, clinical and radiographic features. Results were expressed as mean ± standard deviation for quantitative variables and number (percentage) for qualitative variables. In Univariate analysis difference in means for compliance and non-compliance were performed by Independent Sample t-test, where as difference in proportion were assessed by Pearson Chi-square or Fisher’s exact test where were appropriate. P value less than 0.05 was considered as statistically significant. All p-value were two sided.

## Results

A total of 135 patients were diagnosed to have OSA between 1998 and 2003. At the time of survey, 75 (55.5%) patients (60 males) had a valid contact detail and were contacted. The base line characteristics of patients of OSA with and without valid contact is presented in Table 1.

**Table 1:** **The base line characteristics of patients of Obstructive sleep apnea with and without valid contact**

Characteristics	Valid contact (n = 75)	Without valid contact (n = 60)	P-value
Sex:			
Male	60 (80%)	49 (82%)	0.83
Female	15 (20%)	11 (18%)	
Age (mean) ± SD years	52.9 ± 12.1	50.23 ± 14.0	0.41
BMI	35.89 ± 10.75	36.4 ± 8.23	0.86
Collar size (cm)	35.9 ± 14.4	35.1 ± 11.7	0.9
ESS	15.8 ± 5.6	15.2 ± 8.4	0.87
Smoking status:			
Current smoker	16 (21%)	18 (30%)	
Ex-smoker	19 (25%)	16 (27%)	0.42
Non smoker	40 (54%)	26 (43%)	
**Associated comorbidities**			
Hypertension	42 (57%)	21 (35%)	0.01
Diabetes	21 (28%)	18 (30%)	0.85
Ischemic heart disease	20 (27%)	14 (23%)	0.69
Dyslipidemia	10 (14%)	13 (21%)	0.25
COPD	08 (11%)	10 (16%)	0.32
History of nasal surgery	11 (15%)	08 (13%)	0.99

BMI = body mass index, ESS = Epworth sleepiness scale, COPD = chronic obstructive pulmonary disease.

Out of 75 patients who had a valid contact, continuous positive airway pressure therapy was started by 60 (80%) patients within one month of prescription and 46 (61.3%) continued to use CPAP therapy beyond one year. 56/75 (75%) patients used the machine for over 5 hours each night. CPAP was perceived as a satisfactory treatment by 50 (67%), not satisfactory by 7 (9%) while 18 (24%) were not sure about it. Subjective improvement in snoring was reported by 51 (68%) and in daytime performance by 54 (72%). Objective improvement in Epworth sleepiness scale score was recorded in 54 (72%).

Side effects associated with CPAP therapy were minor but frequent. These included skin changes in 31 (41%), sleep disturbance in 19 (25%), poor mask fit in 15 (20%), eye irritation in 13 (17%), ear symptoms in 9 (12%) and flatulence in 10 (13%).

### Factors associated with compliance

On univariate analysis age, gender, history of snoring, presence of comorbid conditions or smoking status, mask related side effects, OSA severity (as assessed by AHI index) did not affect compliance with CPAP therapy. However, higher body mass index, higher ESS score at baseline, higher prevalence of witnessed apnea, improvements in daytime activity and improvement in ESS score were positively associated with compliance with CPAP therapy, while CPAP induced sleep disturbance was associated with non-compliance with CPAP therapy (Table 2).

**Table 2:** **Factors associated with compliance with CPAP therapy beyond one year in Pakistani patients on univariate analysis**

Factors	Compliant	Non-Compliant	p-value
	(n = 46)	(n = 29)	
**Affecting increasing compliance with**			
**CPAP therapy**			
BMI (kg/m^2^)	38.2 ± 11.9	31.7 ± 6.5	0.015
ESS score before CPAP	17.5 ± 4.1	12.6 ± 6.7	0.005
Witnessed apnea before CPAP	33 (72%)	7 (30%)	0.001
Improved daytime activity with CPAP	43 (94%)	11 (73%)	0.05
ESS score improved with CPAP	43 (94%)	11 (73%)	0.05
**Affecting non-compliance with CPAP therapy**			
Sleep disturbance with CPAP	9 (20%)	10 (67%)	0.001

BMI = body mass index, CPAP = continuous positive airway pressure, ESS = Epworth sleepiness scale.

## Discussion

Privately paying Pakistani patients who had a valid contact (75/135) at the time of study with obstructive sleep apnea had 80% (60/75) initial acceptance of CPAP therapy and 61.3% (46/75) continued to use CPAP therapy beyond one year. This initial compliance rate was similar to that reported in western studies [12]. Long-term compliance with CPAP therapy varied between countries, lower compliance rates were reported in North America compared to Europe [12].

Studies on CPAP compliance in ethnic groups are limited. In a Chinese study, 33% never commenced CPAP therapy [13]. Even lower compliance rate (36%) was observed among bus drivers in Hong Kong despite significant improvement in subjective sleepiness and cognitive function with CPAP therapy [14]. In a study from USA, CPAP adherence was low in black subjects [15]. Similarly Maori subjects had lower adherence to CPAP therapy than Maori subjects that was thought to be related to lower educational levels and socioeconomic status [16]. Thus, differences in compliance with CPAP therapy were multifactorial such as ethnicity, educational and socioeconomic status, and differences in treatment facilities and follow-up.

Differences in compliance may occur in patients being treated in private and public sectors. This aspect of CPAP therapy has not been studied in detail. In a study from Brazil [17], patients in private sector had more greater severity of OSA than patients in public sector (AHI 31 ± 25 vs. 25 ± 24, p < 0.001) but CPAP acceptance was similar in both sectors (32 vs. 35%). This acceptance rate was much lower than that observed in our cohort of private sector patients as well. In Pakistan the health care facilities in public sector are suboptimal and inadequate resources are available to provide top quality healthcare provision. The peoples who seek these facilities are mainly from low socio-economic groups. The sleep study facilities were not available in most of the public sector hospitals of Pakistan at the time of study and they referred all these patients to private sector hospitals. When we did this study not a single public sector hospital offer diagnosis and treatment facility in the city. This is one of the major reason and a limitation that we are unable to compare our cohort with public sector cohort.

In our study the long-term compliance with CPAP therapy was 61% among all OSA patients but 76% among those who initially accepted CPAP. In a study from UK, 77% were still using CPAP at three years and median use of CPAP in those continuing therapy was 5.3 hours/night [18]. In another UK-based study of over 1000 OSA patients, 4.5% patients refused CPAP treatment (these were more often female and current smokers) and during follow-up 20% stopped treatment, primarily because of a lack of benefit. Methods of survival analysis showed that 68% of patients continued treatment at 5 year [19]. The compliance was higher (86%) among those with severe OSA (AHI > 30/hr) accompanied with daytime sleepiness. Among a cohort of 109 German patients treated with CPAP, only 46.6% met stringent criteria for long-term compliance, defined as a mean use of the CPAP machine for at least 5 hours per night [20]. In Canada, using a population-based CPAP program consisting of consistent follow-up, “troubleshooting”, and regular feedback to both patients and physicians CPAP compliance rates of greater than 85% over 6 months was achieved [21]. In our set up, choice of nasal interface was limited, patients often had to travel long distances and the onus of follow-up remained with the patient. Despite these limitations a long-term compliance of above 60% was seen.

Factors associated with compliance with CPAP therapy are manifold. These include patient characteristics, symptoms, psychological factors, sleep study variables, response to therapy, side effects, cost of treatment, expertise available, and use of interventions and educational strategies to improve compliance with treatment. In our study, OSA severity as assessed by AHI did not correlate with a better compliance. Similar findings were reported from USA and UK, in which long-term compliance was not associated with AHI severity but with daytime symptoms [22] or negatively with side effects associated with CPAP therapy [23]. However, other studies have shown a positive association between compliance and severity of OSA as assessed by AHI [24, 25]. Age and gender were not associated with compliance with CPAP therapy in our study, and variable results have been reported in literature [21, 25].

In our study use of anti-depressant was associated poor compliance with CPAP therapy. This was in keeping with other studies showing that psychological factors contributed partly to compliance with therapy [26]. High anxiety and depression score were associated with poor compliance with CPAP therapy [27]. Recent life-events and living alone were also associated with lower machine use [28].

Patients who experience side effects from CPAP machine are less likely to comply with therapy but the nature of side effects that impacts compliance have varied between studies [21, 23, 25]. In our study, sleep disturbance associated with the use of CPAP therapy was strongly associated with poor compliance with therapy.

In our study, long-term compliance with CPAP therapy was better in those patients who achieved an improvement in daytime symptoms. CPAP therapy was perceived as an effective treatment by majority (67%) of our patients, similar to finding from Toronto study where 81% perceived CPAP as an effective treatment [29]. Improvement in ESS score was associated with better compliance in one study [21], but improvement in multiple sleep latency in another study was not associated with higher CPAP usage [23].

There are certain limitations of our study. First there is a small sample size and we have only valid contact of 75 out of 135 patients. The most important reason for this level of dropout is that most of the patients give their cell phone numbers for contact and there is a very common practice and habit of our population that they change their cell phone numbers and services very frequently. As this is a single center study and most of the patients with and without valid contact are from the same background so that we can assume that the level of withdrawal would also be same in patients without a valid contact. Second we use telephone survey to assess the compliance of CPAP therapy. We know that there are some problems for precise evaluation for this method and now newer CPAP machine has built-in-capabilities of keeping record of day and night usage. During the study period these types of machines were not available in Pakistan and almost all of the patients use machines without these facilities. We try to overcome this shortcoming by asking multiple questions on compliance by using a preformed detailed structured questionnaire. Future studies using records of CPAP machine would be more reliable regarding compliance of CPAP usage. We use only univariate analysis with a small sample size is another limitation and studies with larger sample size with logistic regression analysis would be more helpful.

## Conclusion

In conclusion, privately paying Pakistani patients were able to achieve similar level of compliance with CPAP therapy as in other countries. Severity of OSA symptoms at presentation and benefit from treatment predicted higher compliance with CPAP therapy. Severity of OSA as assessed by sleep study did not affect compliance with therapy.
